# Cellular FLICE-like inhibitory protein (cFLIP) critically maintains apoptotic resistance in human lens epithelial cells

**DOI:** 10.1038/s41419-021-03683-y

**Published:** 2021-04-09

**Authors:** Jingru Huangfu, Caili Hao, Zongbo Wei, I. Michael Wormstone, Hong Yan, Xingjun Fan

**Affiliations:** 1 Department of Cellular Biology and Anatomy, Medical College of Georgia at Augusta University, Augusta, GA USA; 2 Department of Ophthalmology, Chongqing Medical University, Chongqing, China; 3 Xi’an Fourth Hospital, Affiliated Guangren Hospital School of Medicine, Xi’an Jiaotong University, Xi’an, China; 4 School of Biological Sciences, University of East Anglia, Norwich, UK

**Keywords:** Tumour-necrosis factors, Apoptosis

## Abstract

The present study aims to understand the mechanism of the lens epithelial cell’s strong anti-apoptotic capacity and survival in the mature human lens that, on the one hand, maintains lens transparency over several decades, while on the other hand, increases the risk of posterior capsule opacification (PCO). Here we compared FHL124 cells and HeLa cells, spontaneously immortalized epithelial cell lines derived from the human lens and cervical cancer cells, respectively, of their resistance to TNFα-mediated cell death. TNFα plus cycloheximide (CHX) triggered almost all of HeLa cell death. FHL124 cells, however, were unaffected and able to block caspase-8 activation as well as prevent caspase-3 and PARP-1 cleavage. Interestingly, despite spontaneous NFκB and AP-1 activation and upregulation of multiple cell survival/anti-apoptotic genes in both cell types, only FHL124 cells were able to survive the TNFα challenge. After screening and comparing the cell survival genes, cFLIP was found to be highly expressed in FHL124 cells and substantially upregulated by TNFα stimulation. FHL124 cells with a mild cFLIP knockdown manifested a profound apoptotic response to TNFα stimulus similar to HeLa cells. Most importantly, we confirmed these findings in an ex vivo lens capsular bag culture system. In conclusion, our results show that cFLIP is a critical gene that is regulating lens epithelial cell survival.

## Introduction

For most people, the mature human lens maintains its transparency for several decades before beginning to increase the risk of developing cataracts^1,2^. Lens epithelial cells (LECs) continue to proliferate and differentiate throughout life, but this rate steadily declines post early development stage^3,4^. The lens epithelium is pivotal to lens transparency by providing nutrients from the aqueous humor to the lens^5,6^, maintaining lens homeostasis through ion channels and transporters^7,8^, and protecting adjacent fibers from stress-mediated damage and aggregation, the key mechanisms of cataractogenesis^9,10^. Lens epithelial cells (LECs) are constantly under insults and stresses, such as UV light, smoking, post-translational modifications, and stressors in the circulating aqueous humor. Several inflammatory cytokines, such as tumor necrosis factor-alpha (TNFα) and interleukins, are detected in the aqueous humor and are elevated under several ocular disease conditions^11,12^. Thereby, lens epithelial cells need to survive challenges from their surrounding environment.

Whether LECs undergo programmed cell death (apoptosis) in the mature human lens with or without cataract has gained much attention in the field. In 1995, Li et al.^13^ reported a stunning 4.4 to 41.8% apoptotic rate of LECs in human cataract lenses based on TUNEL staining. However, in 1998, Harocopos et al.^9^ showed that minimal evidence of apoptotic LECs in both healthy and cataract lenses were detected and the study also suggested that the high level of TUNEL positive stain from the Li et al.^13^ study was likely resulted from necrotic cell death generated during tissue processing. Several follow-up studies also pointed to a minimal level of apoptosis in LECs^14–16^. Preservation of the lens epithelium is vital to maintain transparency, and minimizing cell loss plays an important role in this function. A loss of cells within the epithelium, through apoptosis, would compromise the system. Therefore, a relatively high threshold to apoptosis is beneficial for lens maintenance and protection against cataracts. In contrast, this intrinsic ability to survive becomes problematic following cataract surgery.

During cataract surgery, part of the anterior lens capsule is removed via capsulorhexis, followed by phacoemulsification to remove the fiber mass before intraocular lens (IOL) implantation. However, despite severe disruption to lens integrity, some residual lens epithelial cells always remain following surgery, and these LECs survive, proliferate, migrate, and differentiate. The cellular events subsequently give rise to physical changes, such as matrix wrinkling which scatters light and significantly reduces vision quality^17^. This post-surgical complication of cataract surgery is called posterior capsule opacification (PCO). Therefore, understanding the mechanisms that make lens epithelial cells great survivors and demonstrate a relatively high level of resistance to stress-induced apoptosis will be of great importance in understanding the basis of PCO.

In the present study, we demonstrate a distinct insensitivity of human lens epithelial cells (FHL24) to tumor necrosis factor α (TNFα, a known promoter of apoptosis) apoptosis relative to HeLa cells and report that cellular FLICE-like inhibitory protein (cFLIP) is a pivotal anti-apoptotic gene that is critical for lens epithelial cell survival.

## Results

### Lens epithelial cells are resistant to TNFα-induced cell death

**Fig. 1:**
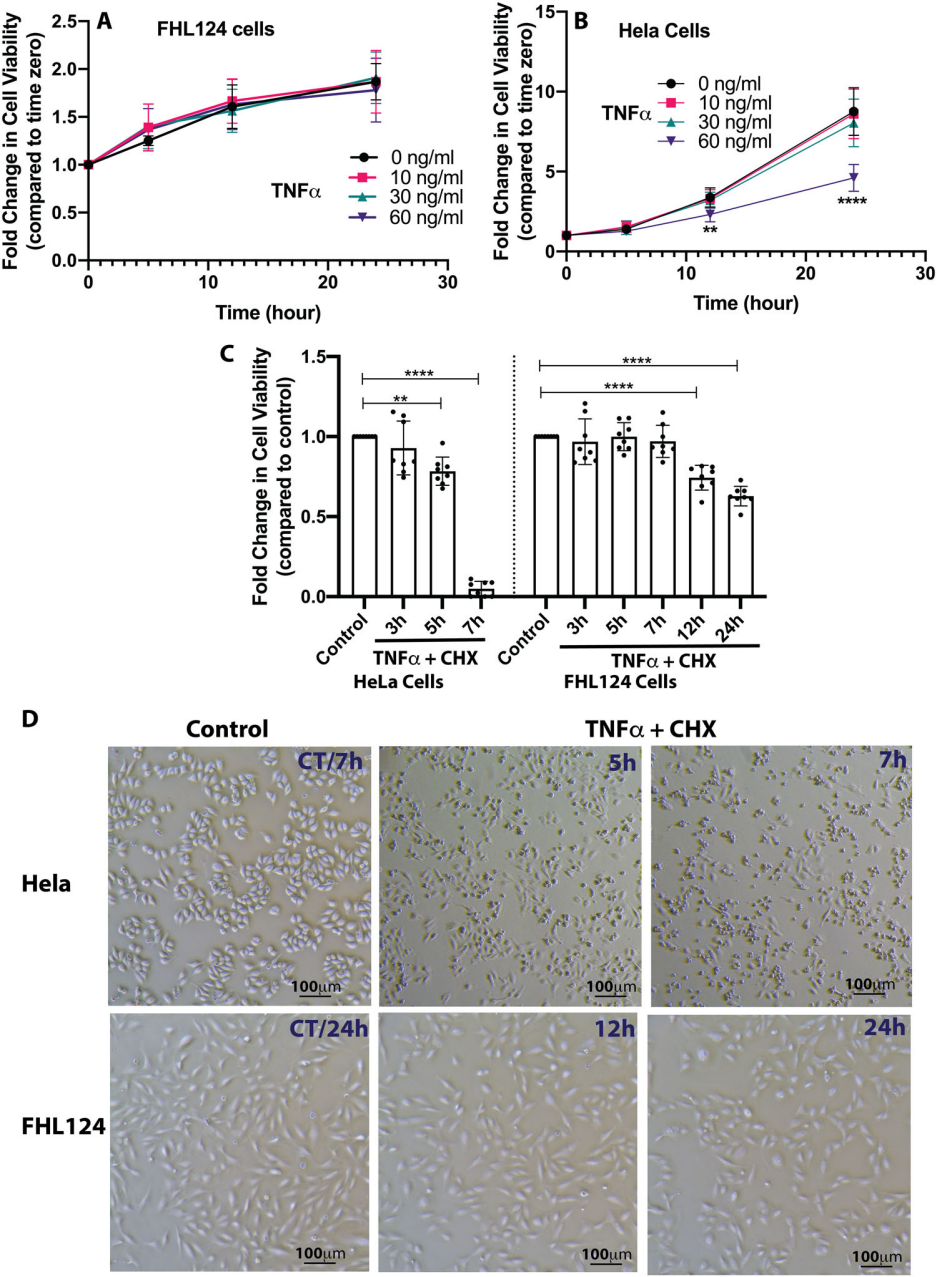
FHL124 but not HeLa cells are resistant to TNFα-induced cell death. **A** FHL124 cells were treated by 0, 10, 30, and 60 ng/ml TNFα for 5, 12, and 24 h. The cell viability was determined by CCK8 assay (*n* = 8). No cell viability loss was seen at any of the TNFα concentrations and time points. **B** HeLa cells were treated with the same levels of TNFα concentrations and time points. Cell viability loss was seen at 12 h and significantly elevated at 24 h. **C** FHL124 and HeLa cells were treated with 30 ng/ml TNFα and 10 μg/ml CHX for multiple time points from 3 h to 24 h (*n* = 8). Profound cell viability loss was seen in HeLa cells 7 h after treatment, but less than 50% cell viability loss was observed in FHL124 cells even after 24 h of treatment. **D** Cell morphology recorded by the phase-contrast imaging showed normal FHL124 cell morphology at 12 h and 24 h time point, but shrunken cell bodies of HeLa cells at 5 h and 7 h time point after TNFα and CHX treatment compared to non-treated cells. One-way ANOVA with Tukey’s Honest post-hoc analysis was used to compare between groups, and only *p* < 0.05 is considered significant. *<0.05, **<0.01, ***<0.001, ****<0.0001.

We used the FHL124 lens epithelial cell line in this study. The FHL124 cell line is a spontaneously immortalized cell line derived from the human lens epithelium^18^. For comparison, we used the HeLa cell line, also a spontaneously immortalized epithelial cell line derived from cervical cancer tissue. To test the cell viability in response to TNFα challenges, both FHL124 and HeLa cells were cultured with 0, 10, 30, and 60 ng/ml TNFα for up to 24 h. As illustrated in Fig. 1A, FHL124 cells proliferated at all TNFα concentrations with no remarkable amounts of cell death. In contrast, at a higher concentration of TNFα (60 ng/ml), HeLa cells demonstrated reduced cell viability starting at 12 h and significantly worsened at 24 h with an approximately 50% reduction compared to non-treated cells (Fig. 1B). To enhance the sensitivity of TNFα-induced cell death, we challenged cells by 30 ng/ml TNFα with the presence of 10 μg/ml cycloheximide (CHX), a protein synthesis inhibitor. As demonstrated in Fig. 1C, TNFα plus CHX triggered ~20% cell death in HeLa cells after 5 h stimulation, and massive cell death (>95%) was observed after 7 h treatment compared to non-treated cells. Surprisingly, FHL124 cells showed a profound resistance to the stimulation. We saw no loss in cell viability after 3 to 7 h of treatment (Fig. 1C). Over 75 and 65% of cells were still viable even after 12 h and 24 h of stimulation, respectively. To better understand FHL124 cells’ stress response mechanism, we extend TNFα and CHX treatment time to 12 and 24 h in the rest of the study.

The cell morphology displayed by phase-contrast imaging at various time points is shown in Fig. 1D–I. HeLa cells demonstrated shrunken cell bodies after 5 h of treatment, and number of shrunken cell bodies increased at a 7 h time point. In contrast, FHL124 cells manifested a normal cell morphology compared to non-treated cells at both 12 h and 24 h time points of the stimulation.

### Lens epithelial cells prevent programmed cell death by blocking caspases activation

**Fig. 2:**
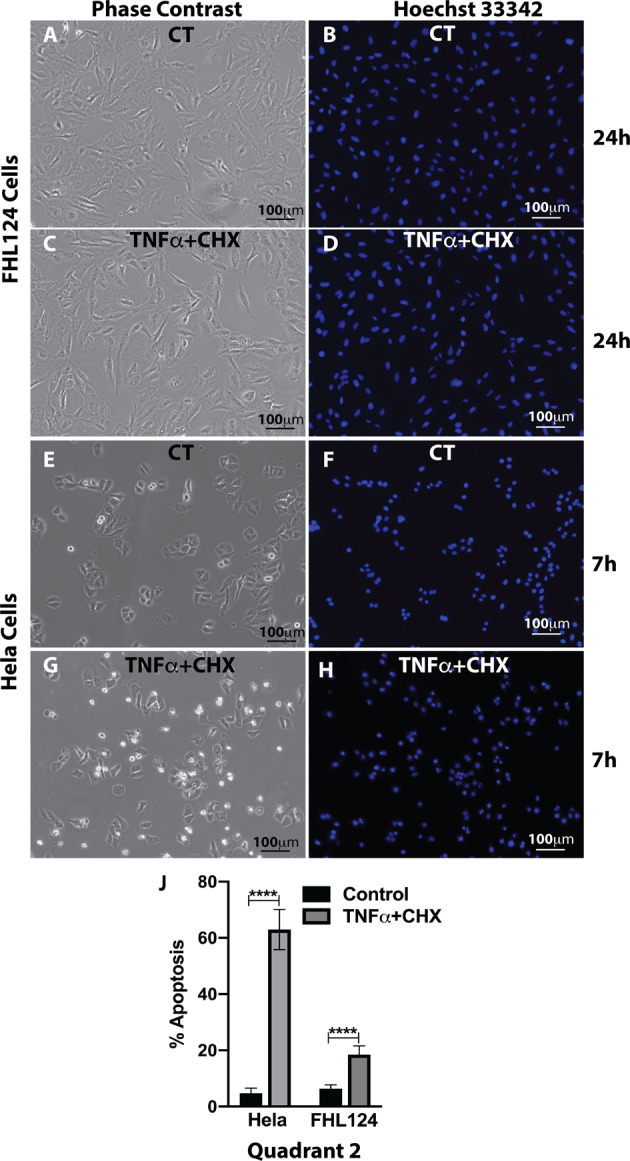
TNFα induces apoptosis in HeLa cells but is resisted by FHL124 cells. **A**–**D** Cell nuclear morphology imaged via Hoechst 33342 stain showed no remarkable changes in FHL124 cells 24 h after TNFα and CHX stimulation. **E**–**H** Nuclear chromatin packaging into apoptotic bodies manifested as condensed nuclei with super bright appearance were seen in HeLa cells 7 h after treatment compared to no treated cells. **J** Active apoptotic cells measured by flow cytometry probed by FITC-Annexin V and propidium iodide (PI) demonstrated significantly increased apoptosis in HeLa cells 7 h after TNFα and CHX stimulation. On the contrary, less than 20% active apoptotic cells were detected 24 h after TNFα and CHX treatment in FHL124 cells. The Student’s *t* test was used to compare treated and no treated cells in each cell type, and only *p* < 0.05 was considered significant. *<0.05, **<0.01, ***<0.001, ****<0.0001.

To further dissect the molecular mechanism of TNFα-mediated cell death, we first examined the cell nuclear morphology by Hoechst 33342 staining using fluorescence microscopy. As shown in Fig. 2E–H, condensed nuclei with a super bright appearance were seen in HeLa cells at both 5 h and 7 h after TNFα and CHX treatment compared to non-treated cells. The condensed nuclei were shown to be from those shrunken cells after comparing the same imaging field’s phase-contrast image (Fig. 2G). In contrast, as shown in Fig. 2A–D, we did not see any condensed nuclei or shrunken FHL124 cells even at the 24 h time point of treatment. These results indicated that the HeLa cells underwent apoptosis because the apoptotic cells are characterized by nuclear chromatin packaging into “apoptotic bodies” with proteins manifesting as nuclear condensation^19^. To further titrate the ratio of apoptosis and necrosis in TNFα- and CHX-mediated HeLa cell death, we stained cells with FITC-labeled annexin V and propidium iodide (PI). As shown in Fig. 2J and Fig. S1, HeLa cells had more than 60% active apoptotic cells (both annexin V and PI-positive, quadrant 2) after 7 h of TNFα and CHX treatment, while FHL124 cells had less than 20% active apoptotic cells after 24 h of TNFα and CHX treatment.

**Fig. 3:**
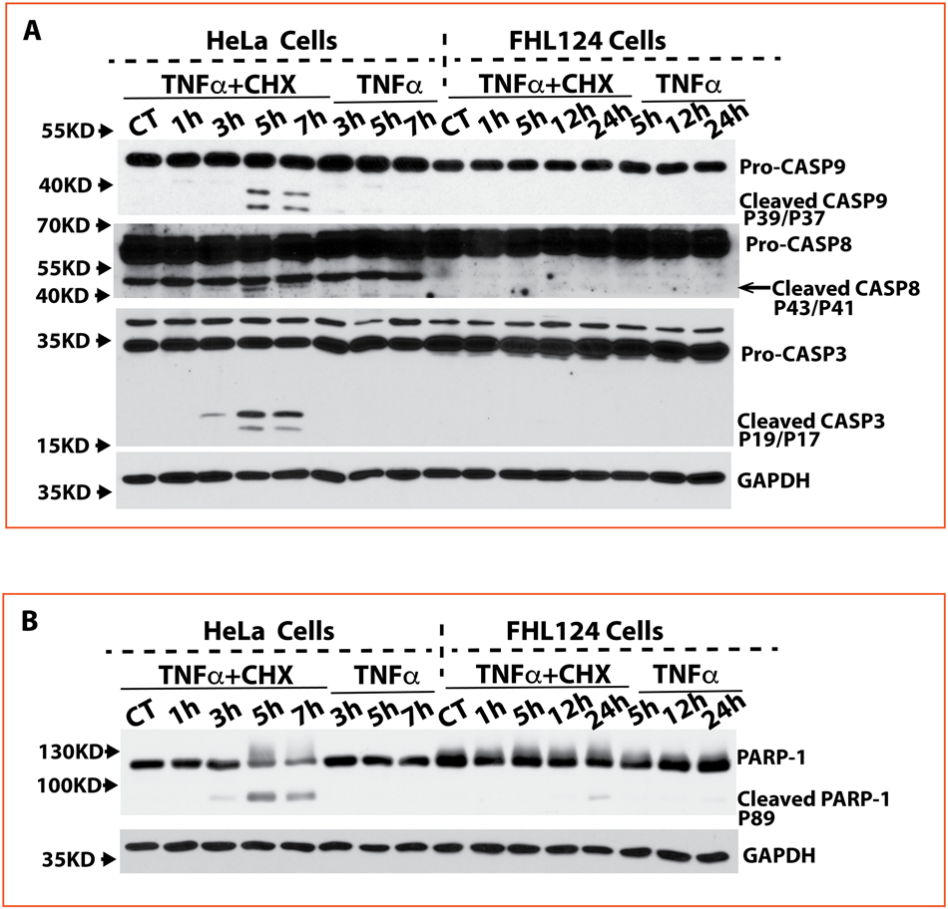
FHL124 cells block caspase 8, 9, and 3 activation and PARP-1 cleavage. **A** Activated caspase-8, caspase-9, and caspase-3 were detected in HeLa cells starting at 3 h and further increased at 5 h and 7 h after TNFα and CHX stimulation. No activated caspase-8, caspase-9, and caspase-3 were detectable in FHL124 cells spanning 24 h of treatment with TNFα and CHX. A higher pro-caspase-9 quantity (around 2-fold) was seen in HeLa cells than in FHL124 cells, but a higher pro-caspase-3 quantity (around 3-fold) was seen in FHL124 compared to that in HeLa cells. **B** Cleavage PARP-1 was detected only in HeLa cells but not in FHL124 cells after TNFα and CHX stimulation.

To further unravel the cascade of TNFα-mediated cell apoptotic pathway in both cell lines, we examined caspase-8, capase-9, and caspase-3 activations by determining their pro- and cleaved forms via immunoblot assay. As shown in Fig. 3A, activation of all caspases was clearly observed in HeLa cells at 5 h, and profoundly exacerbated at 7 h after stimulation compared to non-treated cells. In contrast, no cleaved-caspases were detected in FHL124 cells for up to 24 h challenge (Fig. 3A). We also checked the cleavage of poly (ADP-ribose) polymerase-1 (PARP-1), a DNA repair enzyme, often inactivated by caspase-mediated cleavage during apoptosis^20,21^. As shown in Fig. 3B, PARP-1 cleavage was detected at all three time points in only HeLa cells after TNFα and CHX treatment but was not detectable in FHL124 cells after 24 h stimulation.

### TNFα induces the expression of cell survival genes in both HeLa and FHL124 cells

Next, we want to understand the mechanisms that give FHL124 the leverage to maintain a robust anti-apoptotic capacity over HeLa cells. Using real-time PCR, we screened over 40 genes reported to be closely associated with TNFα signaling (Table 1).

**Table 1:** Relative mRNA expression of associated genes after TNFα and CHX stimulation.

Gene name	FHL124 cells				Hela cells				Endogenous mRNA ratio (FHL124/Hela)
	mRNA fold change after TNFα + CHX treatment*								
Time (h)	5 h	7 h	12 h	24 h	1 h	3 h	5 h	7 h	
TNF-R1		0.68						1.17	1:1.06
TRADD		1.05						0.91	1.45:1
FADD	1.03		0.88	0.96	1.09	0.86	0.87		1:1
TRAF1	147.38		65.76	16.78	5.52	127.91	236.73		5.56:1
TRAF2		2.42						4.10	1.06:1
TRAF5		0.83						0.47	1:2.94
TRAF6	2.17		2.01	2.60	0.77	1.70	2.50		2.78:1
DCR3	5.39		3.53	4.37	6.64	2.87	2.77		3.45:1
RIPK1		nd						nd	
RIPK3		1.09						nd	
FAS/CD95		2.20						1.16	1:4.78
TRAILR1	2.50		5.88	13.63	1.39	1.25	1.61		1:4.83
TRAILR2	6.94		5.80	6.07	2.01	6.93	8.68		1.79:1
NFKB1		11.13						10.17	1:1.14
XIAP		1.71						2.23	1.32:1
cFLIP	6.32		2.94	1.65	1.44	1.94	2.7		5.88:1
BCL2L1	1.15		1.38	1.34	2.43	0.94	1.08		1.22:1
BCL2A1	19.90		4.36	0.99	3.90	10.14	11.30		58.5:1
CIAP1	6.36		3.85	2.05	5.20	7.23	9.93		1.72:1
CIAP2	35.41		21.25	17.52	2.72	15.69	24.08		1:3.32
Survivin		1.15						1.69	2.78:1
cJUN	7.40		2.16	1.54	49.01	76.63	34.88		4.3:1
cFOS	276.81		143.46	51.77	59.63	56.72	34.14		1:14.51
FosB	66.24		15.85	11.37	171.67	220.99	104.44		1.96:1
FRA1	2.77		0.91	0.82	3.74	5.99	6.54		2.13:1
JunB	15.23		4.56	1.74	15.39	29.04	17.14		1:1.46
ATF2		1.54						1.05	1:1.06
ATF3	40.14		11.10	11.20	102.30	230.03	216.73		1.04:1
cMYC	3.89		1.10	0.63	0.85	1.77	2.98		1:3.87
SRC									1:1.55
CAPN2	0.84		0.96	0.44	1.67	1.34	0.27	0.34	2.94:1
CAPN7	1.05		2.24	2.18	1.36	1.07	0.86		1:1.04
CAPNS1		1.27						0.42	1:1.56
CASP9	1.78		2.75	3.37	1.95	3.59	4.04		1:2.32
CASP8	1.18		0.84	0.75	1.25	1.07	1.21		1.06:1
CASP7	1.72		3.91	4.61	1.33	1.08	1.27		1.66:1
CASP6	0.84		1.07	1.58	1.18	0.94	0.86		2.37:1
CASP3	2.67		2.15	1.97	1.31	1.57	2.10		2.32:1
CUL3	0.65		2.01	2.26	1.12	0.55	0.61		1:1.45
CARP1	0.47		0.30	0.16	0.64	0.40	0.29		1:2.40
CARP2		2.82						1.32	1:2

*The standard deviation was removed due to limited space.

First, we investigated known TNFα-mediated cell death signaling molecules, such as the death-inducing signaling complex (DISC) genes. The TNF receptor type 1 (TNFR1), the primary receptor for soluble TNFα, did not present a noticeable change after TNFα and CHX treatment in either cell line (Table 1). Similar results were seen with the TNFR1-associated DEATH domain (TRADD), Fas-associated via death domain (FADD), and pro-caspase-8 (CASP8). We did see increased mRNA expression of several death receptors, such as TNF-related apoptosis-inducing ligand receptors 1 and 2 (TRAILR1 and TRAILR2), decoy receptor 3 (DCR3), and Fas cell surface death receptor (FAS) after TNFα and CHX stimulation. However, these death receptors’ mRNA expression profiles were approximately the same between HeLa and FHL124 cells.

We did not see a remarkable difference between HeLa and FHL124 cells’ endogenous pro-caspase 8 mRNA and protein levels nor a difference in their TNFα- and CHX-induced changes (Table 1, Fig. 3A). HeLa cells had approximately 2-fold caspase-9 mRNA and protein levels (Table 1, Fig. 3) compared to FHL124 cells. However, no significant differences were seen in either cell types after stimulation. FHL124 had an over 2-fold higher endogenous pro-caspase-3 mRNA (Table 1) and protein (Fig. 3) expression compared to HeLa cells. These results suggest that the high levels of apoptosis resistance exhibited by FHL124 cells are not due to low caspase expression but is likely dictated by genes that regulate the fate of caspase activation.

We saw a significant upregulation of signal molecules that initiate NFκB and AP1 activation, such as TNF receptor-associated factors 1, 2, and 6 (TRAF1, TRAF2, TRAF6), cFOS, cJUN, FosB, FRA1, and JunB. However, we did not find major discrepancy between both cell types before and after TNFα and CHX stimulation (Table 1).

**Fig. 4:**
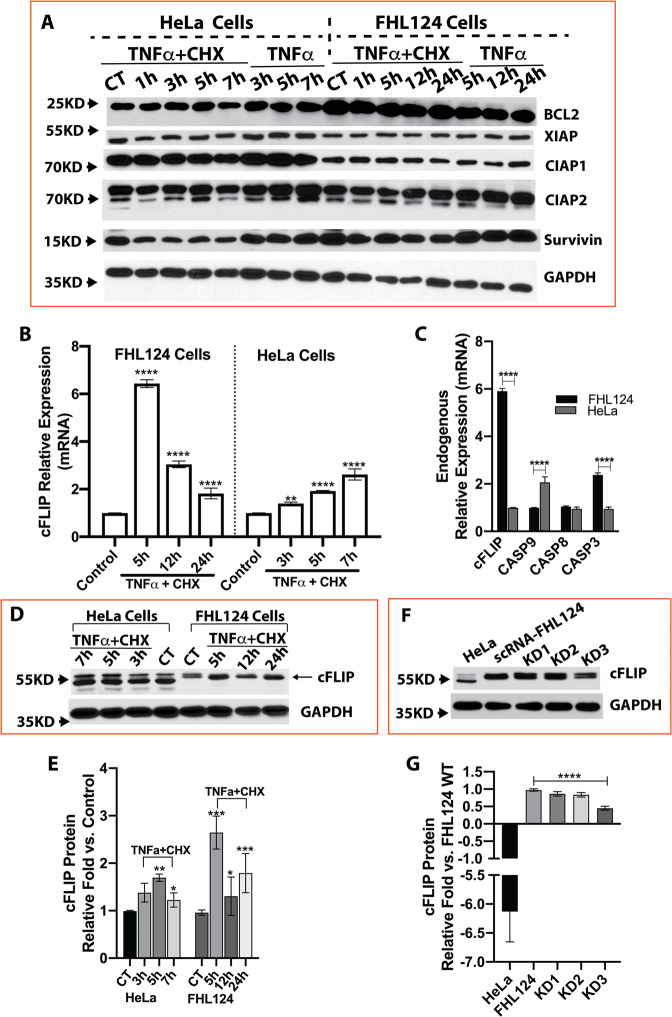
cFLIP is highly expressed in FHL124 cells and also significantly upregulated after TNFα stimulation. **A** Anti-apoptotic protein expression in FHL124 and HeLa cell with and without TNFα and TNFα plus CHX stimulation. **B** Relative cFLIP mRNA expression in FHL124 and HeLa cells with and without TNFα and CHX stimulation. FHL 124 cells demonstrated a more robust response to TNFα and CHX stimulation compared to HeLa cells. **C** The endogenous cFLIP, Caspase-8, caspase-9, and caspase-3 mRNA levels in FHL124 cells vs. HeLa cells. A much higher cFLIP mRNA expression was seen in FHL124 cells compared to HeLa cells. **D**,**E** cFLIP protein expression after TNFα and CHX stimulation in FHL124 and HeLa cells. cFLIP protein level was increased around 3-fold in FHL124 cells at 5 h after TNFα and CHX stimulation, while only a mild increase was seen in HeLa cells after stimulation. **F**,**G** FHL124 cells had 6-fold endogenous cFLIP protein levels vs. HeLa cells. KD3 shRNA was able to knock down 50% of cFLIP expression in FHL124 cells. Each assay was repeated at least three times. One-way ANOVA with Tukey’s Honest post-hoc analysis was used to compare between groups, and only *p* < 0.05 is considered significant. *<0.05, **<0.01, ***<0.001, ****<0.0001.

The robust NFκB and AP-1 activation inspired us to compare various anti-apoptotic genes associated with these two transcription factors. The mRNA expression of Survivin, X-linked inhibitor of apoptosis protein (XIAP), and Bcl-2-like protein 1 (Bclx) was modestly increased (<2-fold), while expression of BCL2-related protein A1 (BCL2A1) and cellular inhibitors of apoptosis protein 1 and 2 (CIAP1, CIAP2) was significantly increased (>2-fold) in both cell lines after TNFα and CHX stimulation. However, no remarkable change was seen in protein expression in both cell lines treated by TNFα with or without CHX (Fig. 4A). Interestingly, TNFα and CHX treatment suppressed CIAP2 and Survivin protein expression in both HeLa and FHL124 cells compared to non-treated cells. Unfortunately, we could not verify the A1 protein expression pattern because of equivocal data obtained by commercial A1 antibodies.

### cFLIP is the critical anti-apoptotic element in FHL124 cells

Cellular FLICE-like inhibitory protein (cFLIP), also known as caspase-8 and FADD-like apoptosis regulator (CFLAR), stood out as a putative candidate. First, the endogenous cFLIP mRNA expression levels in FHL124 cells were approximately 6-fold higher than those in HeLa cells (Fig. 4C). Second, FHL124 cells had a more robust response to treatment via upregulation cFLIP mRNA expression by more than 6-fold while only about 3-fold upregulation was seen in HeLa cells at the 5 h time point (Fig. 4B). The cFLIP protein expression in HeLa and FHL124 cells with and without TNFα and CHX treatment was also determined by immunoblot and was found to match the mRNA expression pattern (Fig. 4D, E).

### A mild degree of cFLIP knocking down triggers FHL124 cells’ apoptotic response to TNFα and CHX

Testing the notion of the critical role of cFLIP in lens epithelial cell apoptosis resistance, we first created cFLIP knockdown FHL124 cells. We screened several shRNA targets and identified two mild knocking down shRNA (KD1 and KD2). We then combined KD1 and KD2 lentiviral particles to create KD3-FHL124-cFLIP knockdown cells with around a 50% reduction of cFLIP protein expression (Fig. 4F, G). The scrambled shRNA (SC) was used as a control.

**Fig. 5:**
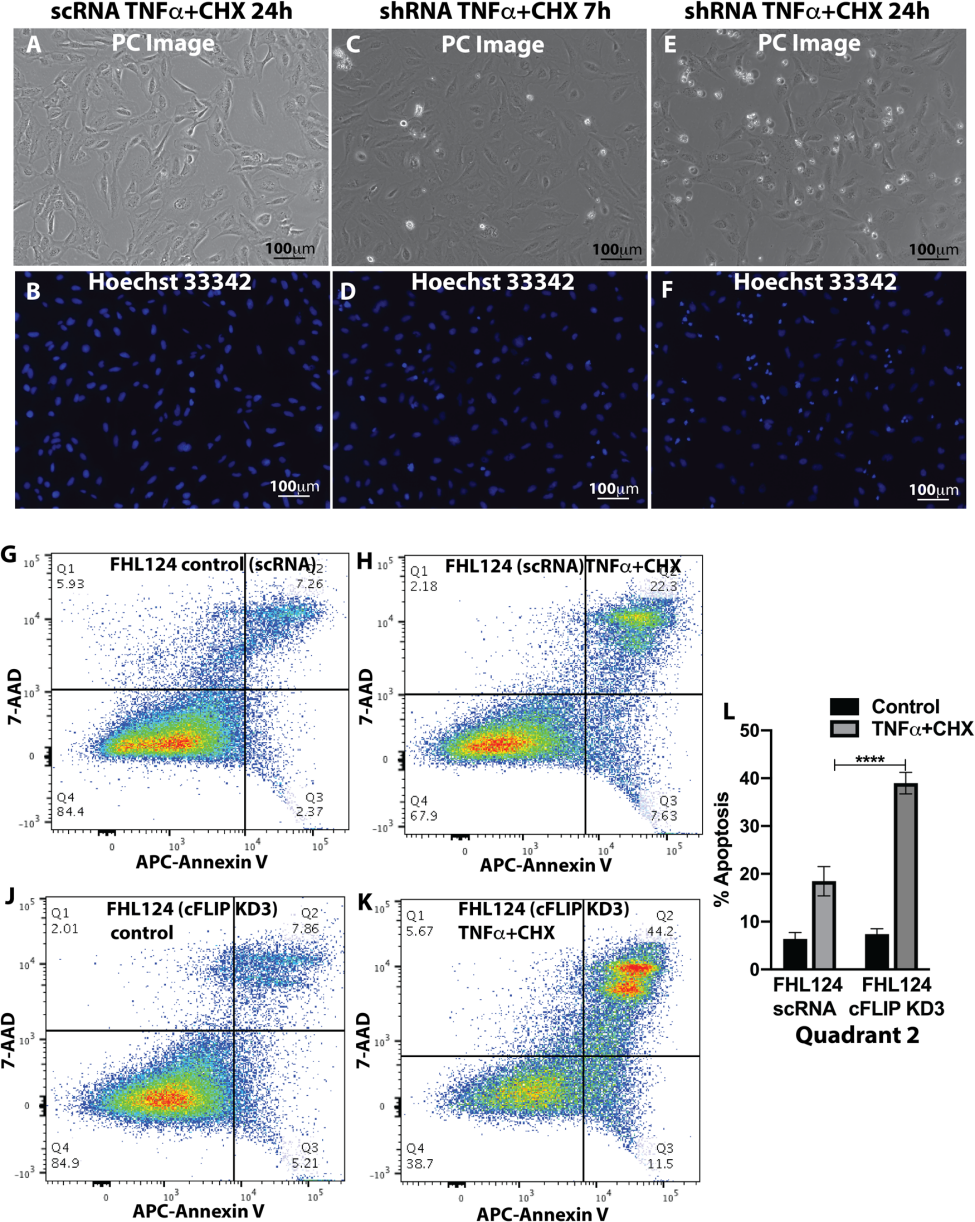
A mild cFLIP knocking down of FHL124 cells manifested with an increased apoptotic phenotype. **A**,**B** Scrambled control shRNA FHL124 cells showed normal nuclear morphology 24 h after TNFα and CHX treatment. **C**,**D** KD3 cFLIP knockdown FHL124 cells demonstrated scattered apoptotic cells after 7 h of treatment. **E**,**F** KD3 cFLIP knockdown FHL124 cells demonstrated a large volume of apoptotic cells after 24 h of treatment. **G**,**H** Active apoptotic cells were analyzed by APC-Annexin V and 7-AAD stain and flow cytometry in scrambled shRNA FHL124 control cells treated with or without 24 h TNFα and CHX stimulation. **J**,**K** Active apoptotic cells were analyzed by APC-Annexin V and 7-AAD stain and flow cytometry in KD3 cFLIP knockdown FHL124 cells treated with or without 24 h TNFα and CHX stimulation. **L** Quantitative data of the ratio of apoptosis in control and cFLIP knockdown FHL124 cells. One-way ANOVA with Tukey’s Honest post-hoc analysis was used to compare between groups, and only *p* < 0.05 is considered significant. *<0.05, **<0.01, ***<0.001, ****<0.0001.

We then tested the cell survival behavior after TNFα and CHX induction. The Hoechst 33342 nuclear stain is shown in Fig. 5, KD3-cFLIP-FHL124 cells demonstrated scattered apoptotic cells (shown as condensed nuclei) after 7 h of stimulation by the same concentration of TNFα plus CHX, and apoptotic cells were significantly increased 24 h after treatment. Minimal numbers of apoptotic cells were detected in SC control cells after 24 h of stimulation. APC-annexin V and the 7-AAD stain were used to determine active cell apoptosis via flow cytometry in cFLIP knockdown and SC FHL124 cells to avoid spectrum overlapping with eGFP introduction during lentiviral-based miR30 shRNA infection. As shown in Fig. 5G–L, mild cFLIP knocking down substantially increased apoptotic cells (>2-fold) after TNFα and CHX stimulation compared to SC control cells.

**Fig. 6:**
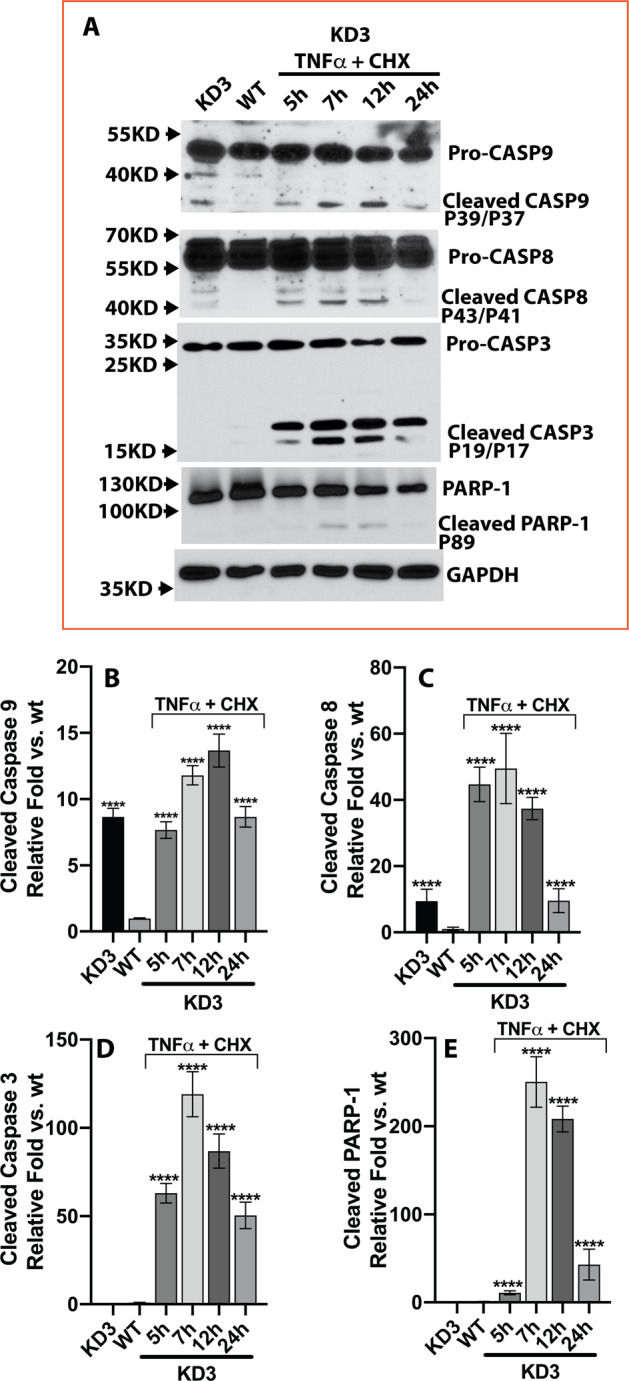
TNFα and CHX trigger an apoptotic response in cFLIP knockdown FHL124 cells by activating caspase-8, caspase-9, and caspase-3. **A** Activated caspase-8 was detected in cFLIP KD3 cFLIP knockdown FHL124 cells from 5 h to 24 h TNFα and CHX treatment. Similar results were seen in activated caspase-9 and caspase-3. PARP-1 cleavage was detected at 7 h and 24 h time point after the stimulation. **B**–**E** The semi-quantitative densitometric measurement. One-way ANOVA was used to compare between groups, and only *p* < 0.05 is considered significant. *<0.05, **<0.01, ***<0.001, ****<0.0001.

Immunoblot analysis also confirmed increased activation of caspase-9, caspase-8, and caspase-3 in KD3-cFLIP-FHL124 cells treated by TNFα and CHX (Fig. 6A–D). We also found increased PARP-1 cleavage at 7, 12, and 24 h after treatment compared to non-treated cells (Fig. 6A, E).

### The crucial role of cFLIP in LECs’ anti-apoptosis is evidenced in an ex vivo mouse lens capsular bag culture

**Fig. 7:**
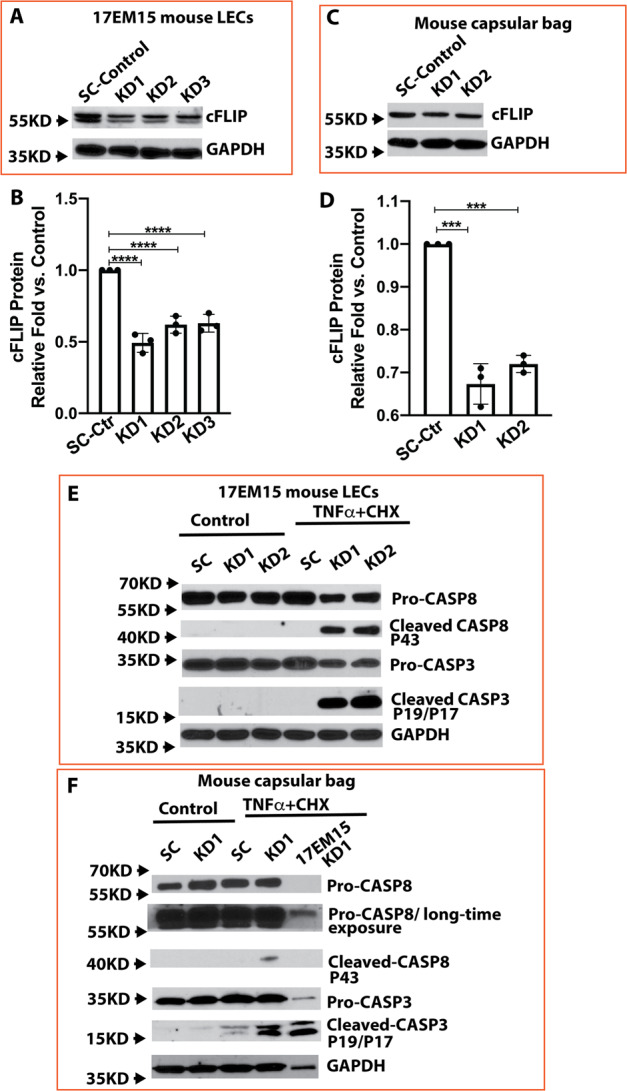
TNFα and CHX trigger an apoptotic response in cFlip knockdown 17EM15 cells and ex vivo cultured mouse lens capsular bag by activating caspase-8, caspase-9, and caspase-3. **A** cFlip knocking down in 17EM15 mouse LECs determined by immunoblot-assay. **B** The semi-quantitative densitometric measurement of 17EM15 cFlip knocking down from immunoblot-assay. **C** cFlip knocking down in ex vivo cultured mouse lens capsular bags. **D** The semi-quantitative densitometric measurement of cFlip knocking down mouse lens capsular bags from immunoblot-assay. **E** Pro-caspase-8, cleaved caspase-8, pro-caspase-3, and cleaved caspase-3 in cFlip knockdown and scrambled shRNA control (SC) 17EM15 cells with and without 30 ng/ml TNFα and 10μg/ml CHX stimulation for 7 h. **F** Pro-caspase-8, cleaved caspase-8, pro-caspase-3, and cleaved caspase-3 in cFlip knockdown and scrambled shRNA control (SC) ex vivo cultured mouse lens capsular bags with and without 60 ng/ml TNFα and 10 μg/ml CHX treatment for 24 h. The cFlip KD 17EM15 treated cell lysate was used as a positive control. Only 1/10th amount of protein relative to capsular bag lysate was loaded. One-way ANOVA was used to compare between groups, and only *p* < 0.05 is considered significant. *<0.05, **<0.01, ***<0.001, ****<0.0001.

All the strong evidence above was resulted from in vitro cell line studies, which raises the inevitable question of whether this is occurring in an in vivo situation. We decided to test the lens epithelium using mouse lens capsular bag culture, an ex vivo model that closely mimics the in vivo situation. We first examined the TNFα-mediated cell responses in 17EM15 cells, an immortalized mouse lens epithelial cell line, to confirm whether human and mouse lens epithelial cells have similar cFLIP empowered anti-apoptotic capacities. A stable cFlip knockdown 17EM15 cell line with ~50% reduction of cFLIP protein expression was established as illustrated in Fig. 7A. As shown in Fig. 7E, remarkable amount of activation of both caspase-8 and caspase-3 were observed in cFlip KD 17EM15 cells, while no detectable amounts of caspase-8 and 3 activation were seen in SC cells after TNFα and CHX treatment. Evidently, both human and mouse lens epithelial cells are highly resistant to TNFα-induced cell death, and cFLIP is the key anti-apoptotic gene in human and mouse LECs.

**Fig. 8:**
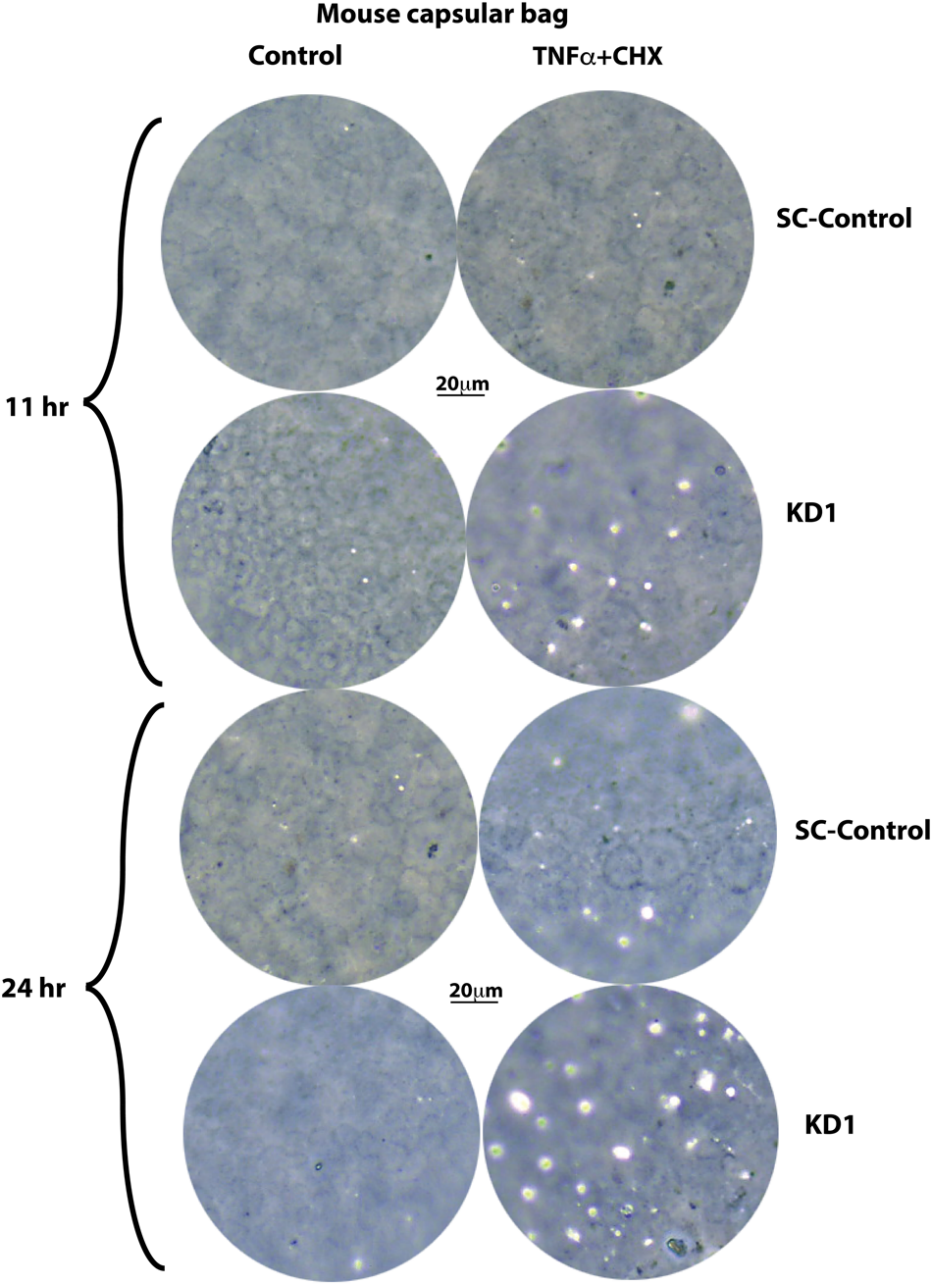
Phase-contrast images of mouse lens capsular bags with and without TNFα and CHX stimulation. (Left panel) Non-treated capsular bags. (Right panel) 60 ng/ml TNFα and 10 μg/ml CHX-treated capsular bags. (Upper parentheses) 11 h treatment with medium (control) or TNFα plus CHX. (Lower parentheses) 24 h treatment with medium (control) or TNFα plus CHX. 10x objective was used for image capture, and *n* = 6/group.

Next, we established an ex vivo lens capsular bag culture and cFlip knockdown lens epithelium model using KD1, KD2, and scrambled shRNA control lentiviral particles. As shown in Fig. 7B, despite our extensive efforts at optimizing the knocking down conditions, KD1 and KD2 could suppress around 30 and 25% of cFLIP protein expression, respectively, compared to the scrambled control (SC) virus. The low efficiency of cFLIP protein knocking down in lens capsular bags should be expected since we cannot select lens capsular bag culture by puromycin. To increase the cell death rate, we treated the lens capsular bag with a double amount of TNFα (60 ng/ml) while keeping the same amount of CHX. The phase-contrast images of the lens capsular bags are shown in Fig. 8. The cuboidal shaped lens epithelial cells were observed although some regions were out of focus due to a ball-shaped capsular bag. Remarkably, the cFlip KD capsular bags demonstrated a considerable cell death at the 11 h time point, and massive cell death was observed 24 h after treatment. In contrast, no apparent cell death was observed in SC capsular bags at the 11 h time point, and only mild cell death was seen at the 24 h time point following treatment. The lens epithelium cell death in capsular bags with cFlip knocking down was also evidenced in cellular caspase-8 and caspase-3 activation. As shown in Fig. 7F, cleaved caspase-8 and 3 were seen in cFlip KD capsular bags, but only weak cleaved caspase 3 bands and no cleaved caspase 8 were detected in SC capsular bags 24 h after TNFα and CHX treatment.

## Discussion

Cell fate, especially live or die, is an essential biological process that critically impacts development, tissue homeostasis, and disease^22^. For example, during early eye development, programmed cell death/apoptosis is required to separate the lens vesicle from the surface ectoderm^9,23,24^. At the later developmental stage, during fiber cell differentiation and lens formation, apoptosis suppression is required^25^. A growing consensus suggests that a minimal level of apoptosis is present in healthy and cataractous human lenses despite various stresses. However, the mechanisms of the strong anti-apoptosis capability and survival rate of the lens epithelial cell in the mature human lens have not been well studied.

In the present study, we confirmed that LECs could survive TNFα stimulation without any cell viability loss at up to 60 ng/ml concentration for 24 h. Multiple cell survival genes were activated following TNFα stimulation. The cellular FLICE-like inhibitory protein (cFLIP) gene was the critical anti-apoptotic gene with high endogenous expression and significant upregulation in the FHL124 cells compared to that in HeLa cells. The FHL124 cells with a mild cFLIP knockdown manifested with a similar apoptotic response as HeLa cells after TNFα and CHX stimulation. Most importantly, these findings were also explicitly confirmed by the ex vivo lens capsular bag culture system, which strongly implicated the crucial role of cFLIP in lens epithelial cell survival. Our present study also suggests that different cell types may have distinct regulatory mechanisms in cell survival.

TNFα, a proinflammatory cytokine, induces the cellular immune response and regulates cell apoptosis and necrosis via the “extrinsic” mediated programmed cell death pathway. TNFα, through interactions with its receptors, such as TNFR1, recruits several adaptor proteins, such as FADD and TRADD^26^. These adaptor proteins will form a death-inducing signal complex (DISC) by recruiting and subsequently activating caspase-8. The activated caspase-8 can either directly trigger cell apoptosis by activating caspase-3, or via BID cleavage, activate the intrinsic apoptotic pathway through first activating caspase-9 and then caspase-3^27^. We found very similar endogenous TNFR1, FADD, and TRADD mRNA expression and their responses to TNFα stimulation between FHL124 and HeLa cells, which implicates that both cell types have similar signaling initiation by TNFα. Moreover, we saw relatively similar caspase-8 expressions in both mRNA and protein levels within HeLa and FHL124 cells. Interestingly, studies have also found that retinal pigment epithelial cells (RPEs) are resistant to TNFα-induced cell death^28,29^. A later study by Yang P. et al.^30^ concluded that a very low endogenous caspase-8 expression is a key underlying mechanism in RPEs’ resistance to TNFα-induced apoptosis. In fact, in the same study, a significantly higher level relative to RPEs (~30-fold) of caspase-8 expression in both mRNA and protein were found in LECs^30^. This suggests that an entirely different mechanism is responsible for LECs’ resistance to TNFα-mediated cell death. Furthermore, FHL124 cells have a much higher pro-caspase-3 expression as compared to HeLa cells. Thus, to protect FHL124 cells from apoptosis, tight regulation of initiator caspases activation, i.e., caspase-8 and 9, is required.

**Fig. 9:**
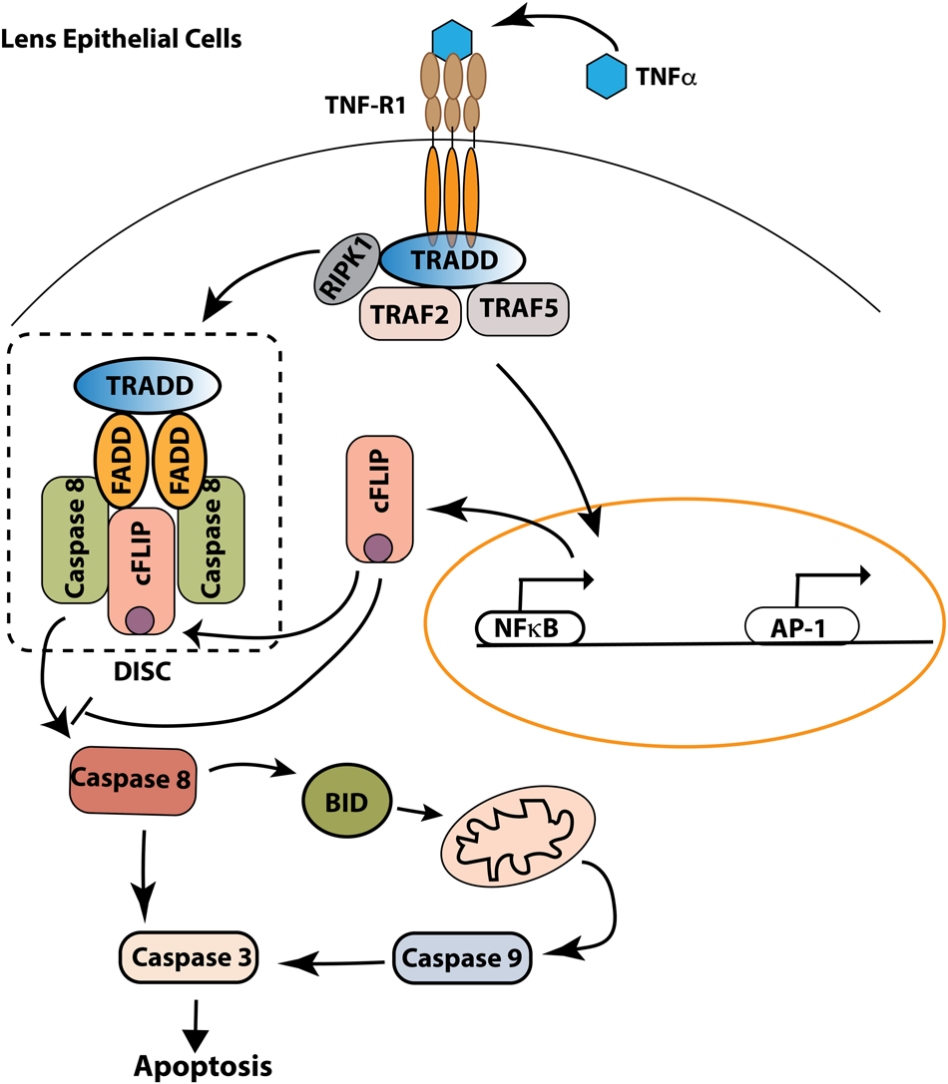
A model of cFLIP-mediated LEC’s survival. A proposed model is highlighting cFLIP regulation of lens epithelial cells’ survival and anti-apoptotic function.

Intriguingly, the cFLIP protein stood out as a critical anti-apoptotic gene in the FHL124 cell response to TNFα-mediated cell death. The cFLIP protein is a pro-caspase-8 protein, and two major cFLIP isoforms are often seen intracellularly originating from mRNA splicing, a 26KD short-form (cFLIPs) and a 55KD long-form (cFLIP_L_). cFLIP_L_ is closely reminiscent of pro-caspase 8 but lacking caspase activity. Growing evidence demonstrates that FADD and pro-caspase 8 can recruit cFLIP_L_ into DISC so that heterodimerization between pro-caspase-8 and cFLIP_L_ replaces pro-caspase-8 homodimerization^31–33^. The cFLIP_L_ incorporation and pro-caspase-8 homodimerization disruption blocks caspase-8 activation^32,34^. In the present study, the cFLIP antibody could only pick up 55KD isoforms (cFLIP_L_), and we could not exclude the possibility that short form cFLIP (cFLIPs) were also present in the FHL124 cells. However, this will not impact the LEC’s strong anti-apoptotic character since both short- and long-form cFLIP can prevent caspase-8 activation^35^. Thus, as illustrated in Fig. 9, we believe that an abundant endogenous cFLIP expression and a robust upregulation upon TNFα stimulation in LECs, synthesizes a sufficient amount of cFLIP protein, which then incorporates into DISC to block caspase-8 activation and apoptotic/necrotic-based cell death. The abundant A1 expression suggests that FHL124 cells may also be resistant to intrinsic pathway-mediated apoptosis. The primary function of BCL2 family proteins in preventing apoptosis is to maintain mitochondrial membrane integrity by blocking BAX/BAK oligomerization and apoptotic molecules’ release, such as cytochrome c^36^. Our present study focused on the TNFα-mediated cell death pathway, an extrinsic cell death pathway. Future studies are needed to study the LEC’s anti-apoptotic and survival capacity when triggered by an intrinsic pathway-mediated apoptotic mechanism.

Posterior capsule opacification (PCO) that arises from surviving residual LECs is also a testament to the lens epithelial cell’s toughness and strong survival rate^17^. Previous work has shown that human lens epithelial cells residing on their natural matrix following cataract surgery on donor lenses in a laboratory setting can survive in a serum-free culture for more than 1 year^37^. Preventing PCO formation by either blocking LEC proliferation or inducing LEC’s apoptosis has gained significant interest in the PCO research field by using cytotoxic drugs, and immunosuppressive agents, such as 5-fluorouracil (5-FU)^38,39^. Although these pharmacological interventions saw some level of restraint in PCO formation, the impact of these drugs’ cytotoxicity to other ocular tissues is of a significant concern and must be carefully considered^40^. Our findings from this study, especially the mouse lens capsular bag culture study, suggest that even higher dosages may be required to stem LEC growth and PCO formation. More studies are needed to understand whether cFLIP is upregulated in LECs when treated by these drugs. However, by targeting the anti-apoptotic regulatory gene in LECs, such as cFLIP, we may selectively kill LECs and avoid harming other ocular tissue. For instance, RPE cells’ low caspase-8 expression would not be affected by targeting cFLIP.

In summary, the present study reveals that the cFLIP gene is playing a pivotal role in regulating lens epithelial cell survival by preventing caspase-8 activation and subsequent programmed or necrotic cell death pathway. The LEC’s strong survival rate and anti-apoptotic property may explain the mature human lens’s low apoptotic phenomena in the epithelium. This discovery may also suggest cFLIP as a novel therapeutic target for PCO prevention.

## Materials and methods

### Reagents

All chemicals used were of analytical reagent grade. Milli-Q water was used for the preparation of standards and reagents. TNFα (Cat. H8916), cycloheximide (CHX; Cat. C7698), and all other chemical reagents were from Sigma-Aldrich (St. Louis, MO). Hoechst 33342 (Cat. No. H3570) was ordered from ThermoFisher (Waltham, MA).

### Animal

All animal experiments were performed in accordance with procedures approved by the Augusta University Animal Care and Use Committee and conformed to the Association for Research in Vision and Ophthalmology Statement for the Use of Animals in Ophthalmic and Vision Research. C57BL/6 mice were ordered from Jackson laboratory. Animals were housed under a diurnal lighting condition and allowed free access to food and water. The statistical analysis power determined the number of mice used for each assay, and mice were allocated to each experimental group randomly except for gender and age.

### Cell culture and treatment

The human lens epithelial cell line of FHL124 cells^18^, established by Prof. John Reddan at Oakland University, were supplied by Prof. Michael Wormstone at the University of East Anglia, UK and were grown in Minimum Essential Media (MEM) with 5% fetal bovine serum, 2 mmol/L glutamine, and 50 U/ml penicillin/streptomycin (HyClone, Cytiva) at 35 °C in a humidified 5% CO_2_ incubator. The mouse lens epithelial cell line of 17EM15 cells were provided by Dr. Salil Lachke at the University of Delaware. 17EM15 cells, HeLa cells, and HEK293T cells were grown in Dulbecco’s Modified Eagle Medium (DMEM) with 10% fetal bovine serum, 2 mmol/L glutamine, and 50 U/ml penicillin/streptomycin (HyClone, Cytiva) at 37 °C in a humidified 5% CO_2_ incubator. All used cells have been tested free of mycoplasma contamination. FHL124, 17EM15, and HeLa cells (5 × 10^5^ cells/dish) were seeded 16 h before treatment in a 60 mm culture dish. Cells were treated by a freshly prepared full medium containing 0, 10, 30, and 60 ng/ml TNFα, an established trigger for apoptosis. Cells were harvested at 1, 3, 5, 7, 12, and 24 hr time points for analysis. For TNFα and CHX treatment, freshly prepared medium including 30 ng/ml TNFα and 10 μg/ml of CHX were used, and cells were then harvested at various time points as described in each assay. For the mouse lens capsular bag culture, 60 ng/ml TNFα and 10 μg/ml CHX were used.

### Cell viability assay

Cell viability was measured via a colorimetric assay using Cell Counting Kit-8 assay (Cat. HY-K0301, MedChem Express, Monmouth Junction, NJ) following the manufacturer’s instruction. In brief, 7 × 10^4^ cells per well were placed in a 96-well-plate 16 h before treatment. At each experimental time point, 10 µl of CCK-8 solution were added to each well, and cells were incubated at 35 °C and 37 °C for an additional 1.5 h before recording the absorbance at 450 nm. The cell-free medium served as a blank. Cell viability data for each given treatment was expressed relative to the non-treated cells.

### Cell apoptosis analysis by determining the nuclear morphology

Cells were seeded in 60 mm culture dishes at a density of 1.8 × 10^5^ cells/dish 16 h before treatment. To avoid apoptotic cells detaching from the culture dish during the staining and washing procedure, a low concentration (100 ng/ml) of Hoechst 33342 was added at the same time along with TNFα and CHX. At each time point of treatment, the phase contrast image and Hoechst 33342 stained nuclear image were captured without replacing the culture/treatment medium by an EVOS fluorescent microscope (ThermoFisher) with a 10x objective.

### Cell apoptosis analysis by Annexin V and propidium iodide or 7-AAD stain

Cells were seeded in 60 mm culture dishes at a density of 1.8 × 10^5^ cells/dish 16 h before treatment. Since this study focused on cell apoptosis/necrosis, to avoid dead cells being washed away, the cell culture medium and PBS washing buffer were all collected and combined with trypsinized cells. Following a 5 min spin at 300 *g*, the resulting pellet was washed once by 2 ml of cell staining buffer (Cat. 420201, BioLegend, San Diego, CA). The cell pellet was resuspended in 100 μl Annexin V binding buffer (Cat. 640930, BioLegend) and stained by either FITC-Annexin V/Propidium iodide (PI) or an APC-Annexin V/7-AAD combination for 15 min at room temperature in the dark before adding an additional 400 μl binding buffer. The APC-Annexin V/7-AAD combination was used for cFLIP expression knockdown cells, in which eGFP was used as a probe for lentiviral-based shRNA infection. Cell apoptosis/necrosis was analyzed by CytoFLEX (Beckman Coulter) and Attune NxT (ThermoFisher) flow cytometry. All flow cytometry data were analyzed by FlowJo (version 10.7).

### RNA extraction and real-time PCR analysis for gene expression

A 60 mm dish of cultured cells was used for RNA extraction. Cells were treated as described above. At each time point, cells were washed three times with 5 ml ice-cold PBS, and then 1 ml TRIzol reagent (Cat. 15596026, ThermoFisher) was added. The total RNA was extracted following the manufacturer’s instructions. The RNA concentration and quality were determined by DS-11 FX + NanoDrop. Only RNA samples with UV 260 nm/280 nm ratio range of 1.9–2.0 and UV 260 nm/230 nm ratio >1.8 were used. The total RNA was treated with DNase I (Cat. 18047019, ThermoFisher) to remove any trace of DNA being reverse transcribed to complementary DNA with an oligo(dT) random primer mix (Cat. S1330S, New England Biolabs, Ipswich, MA) and M-MuLV reverse transcriptase (Cat. M0253S, New England Biolabs). Real-time PCR was performed using the SYBR Green probe and a QuantStudio 3 Real-Time PCR system (ThermoFisher). Relative expression was calculated using the ΔΔCt method normalized to the housekeeping gene GAPDH. Specific primer sequences are listed in Table S1. All reactions were performed in triplicate. The minimum Information for Publication of Quantitative Real-Time PCR Experiments (MIQE) guidelines^41^ were followed for all real-time PCR experiments.

### Ex vivo mouse lens capsular bag culture

3-month-old mice were used in this experiment. To prepare the mouse lens capsule bag culture, the mouse lens was dissected, and the peripheral tissue attached to the lens was removed. The lens was then washed three times with 10 ml HBSS before being mounted half-way into 2% ultrapure low melting agarose (Cat. 16520050, ThermoFisher) MEM mounting medium in the orientation of the posterior side face up in a 3.5 cm culture dish. A small nick was made at the lens capsule’s posterior pore, 4 to 6 flaps were peeled from the posterior pore to the equator of the lens with small tweezers. The fiber mass was then removed by hydrodissection. The capsular bag was then gently washed twice with HBSS and cultured in MEM containing 100 U/ml of penicillin and streptomycin and 2.5 µg/ml amphotericin B at 37°C in a humidified 5% CO_2_ incubator for 16 h before treatment. 6 capsular bags (*n* = 6) were used in each assay.

### Capturing the lens capsular bag image after treatment

The lens capsular bag’s phase-contrast image was captured by the Leica DMi1 inverted microscope equipped with a 5-megapixel color camera. The 10x objective was used for all imaging capture.

### shRNA-based cFLIP gene knocking down

The shRNA was produced through a miR30-based system using the MSCV-miR30-eGFP vector^42^ for stable knockdown cells. The miR30 vector TM30 and lentiviral packing vectors (VSV-G, BH10 *ϕ*^*−*^*env*^*−*^, pRev) were kindly provided by Dr. Jacek Skowronski at Case Western Reserve University. Two sets of shRNA targeting cFLIP and one set of scrambled shRNA were used for FHL124 cells. KD1: GAGATACAAGATGAAGAGCAAG, KD2: CAGAATAGACCTGAAGACAAAA, Scramble: CTCCCGTGAATTGGAATCC. Two sets of shRNA targeting cFLIP and one scrambled shRNA were used for 17EM15 and ex vivo mouse lens capsule explants. KD1: CCTCACCTGGTTTCTGATTAT, KD2: CCAAGGAGCAAGATCAAATAT, Scramble: CTCCCGTGAATTGGAATCC.

The miR30 shRNA lentiviral particles were produced from HEK293T cells as previously described^43^. FHL124 and 17EM15 cells were infected with mir30 shRNA lentivirus for 48 h, and the stable RNAi cells were grown out of 0.2 μg/ml puromycin selection. For the ex vivo cultured mouse lens capsular bag, shRNA lentiviral particles were added in the culture medium for 48 h, the medium was then removed, and the capsular bag was gently washed once with 2 ml MEM medium before treatment. The cFLIP knockdown was validated by immunoblot analysis (described below).

### Protein extraction and immunoblot assay

Cells were washed twice with ice-cold PBS and lysed by the cell lysis buffer containing 20 mM Tris-HCl pH 7.5, 150 mM NaCl, 1 mM Na_2_EDTA, 1 mM EGTA, 1% Triton, 2.5 mM sodium pyrophosphate, 1 mM β-glycerophosphate, 1 mM Na_3_VO_4_, 1 µg/ml leupeptin, and freshly added 1 mM PMSF. The cells were lysed on ice for 20 min before 10 min 15,000 rpm centrifugation at 4 °C for 15 min. The protein concentration in the supernatants was measured by BCA protein assay (ThermoFisher). Depending on the target protein level and antibody sensitivity, 5–20 μg protein samples were loaded and separated by SDS-PAGE. The separated proteins were then transferred to the PVDF membrane (Cat. IPVH00010, MilliporeSigma, Burlington, MA). The membrane was first blocked by 5% non-fat milk tris buffer saline plus tween 20 (PBST) for an hour at room temperature and then probed with primary antibodies of caspase-8 (Cat. 645501, Biolegend), pro-caspase 8, mouse-specific (Cat. 4927, Cell Signaling, Danvers, MA), caspase-9 (Cat. 9502, Cell Signaling), cleaved caspase 8, mouse-specific (Cat. 8592, Cell Signaling), caspase-3 (Cat. 74T2, ThermoFisher), cleaved caspase-3 (Cat. 9664, Cell Signaling), PAPR-1 (Cat. 13371-1-AP, Proteintech), cFLIP (Cat. No. 10394-1-AP, Proteintech, Rosemont, IL), cFLIP for mouse cells and tissue (AF821, R&D System, Minneapolis, MN), Survivin (Cat. 1058-1-AP, Proteintech), CIAP1 (Cat. 66626-1-AP, Proteintech), CIAP2 (Cat. 24304-1-AP, Proteintech), XIAP (Cat. 10037-1-lg, Proteintech), GAPDH (Cat. PA1-987, ThermoFisher) overnight at 4 °C. The washed membrane was then probed with appropriate HRP-conjugated secondary antibodies (Jackson ImmunoResearch, West Grove, PA) for 1 h at room temperature. Immunoreactive bands were visualized using enhanced chemiluminescence (ECL) substrate (Cat. PI34580, ThermoFisher). The housekeeping gene GAPDH was used as a reference protein, and all target protein expression levels were semi-quantitatively determined by GAPDH adjustment using ImageJ software.

### Statistics

All values are expressed as mean ± SD. In brief, the Student’s *t* test and one-way ANOVA with Tukey’s Honest post-hoc analysis was computed using SPSS and GraphPad 8 software. Testing for homogeneity of variance was done using either the F-test or the Burr–Foster Q-Test, as previously described^44^. Significance was considered *P* < 0.05.

## Supplementary information

supplemental material
