# Supplementary material for: Cellular FLICE-like inhibitory protein (cFLIP) critically maintains apoptotic resistance in human lens epithelial cells
Source: Cell Death Dis. 2021 Apr 9;12(4):386. doi: 10.1038/s41419-021-03683-y (PMC8035156; doi:10.1038/s41419-021-03683-y)
Supplement: Supplementary file 1 — supplemental material [file 41419_2021_3683_MOESM1_ESM.pdf]

Table S1 Real-time PCR primers used in the study

Gene Name	Sense primer	Anti-sense primer
TNF-R1	GTGCTGTTGCCCCTGGTCAT	GCTTAGTAGTAGTTCCTTC
TRADD	GGACCCTGAAACTCCACTTG	GATGAAGTCCAGGACACCAA
FADD	CAGAGCCCATGCTCAACC	ACATCCTTTCTGCCAACTGC
TRAF1	CCG GAA CAA GGT CAC CTT CAT GC	TGG GCA TCC ACT GGC CAC G
TRAF2	GGC CCT TCA ACC AGA AGG TGA CC	CGA TGT TCA TGT CGT TGA CTG GC
TRAF5	CGGTACCAGGATCACCTTCA	GTCTTTCCGTAGGACTGGCT
TRAF6	CTGCTTGATGGCATTACGAGAA	TGCAGGCTTTGCAGAACCTA
DCR3	TCAATGTGCCAGGCTCTTC	GCCTCTTGAT GG AGATGTCC
RIPK1	TCCTGGGCGTCATCATAGAG	CGGAGTACTCATCTCGGCTT
RIPK3	AGAAGTGGTGCTTGGGATG	AAATCTGCCAGCTTGACGTG
FAS/CD95	ACCCTCCTACCTCTGGTTCT	GATGCAGGCCTTCCAAGTTC
TRAILR1	CCCACTGAGACTCTGATGCT	CTGTACCAGCTCTGACCACA
TRAILR2	GGACAGAAGCTCACAACGAC	ACATGTTGACACCTGTTGGC
NFKB1	CCT GGA TGA CTC TTG GGA AA	CTA GCC AGC TGT TTC ATG TC
XIAP	GGTGTTTTCTCAGTAGTTCTTACCAGACA	ATGCTAAATGGTATCCAGGGTGC
cFLIP	ATT GCA TTG GCA ATG AGA CAG AGC	TCG GTG CTC GGG CAT ACA GG
BCL2L1	GCA GGT ATT GGT GAG TCG GAT CGC	CAC AAA AGT ATC CCA GCC GCC G
BCL2A1	AAA TTG CCC CGG ATG TGG ATA CC	TTT CCC AGC CTC CGT TTT GCC
CIAP1	AGC CTG AGC AGC TTG CAA GTG C	CCC ATG GAT CAT CTC CAG ATT CCC
CIAP2	CCG TCA AGT TCA AGC CAG TTA CCC	AAG CCC ATT TCC ACG GCA GC
Survivin	ATT CGT CCG GTT GCG CTT TCC	CAC GGC GCA CTT TCT TCG CAG
cJUN	CGTGAAGTGACGGACTGTTC	GTAGCCATAAGGTCCGCTCT
cFOS	TACACTCCAAGCGGAGACAG	ATCAAGGGAAGCCACAGACA
FosB	GTG TGA GCG CTT CTG CAG C	CCA ATT CAA CGG CTC GCT T
FRA1	GCCTTGTGAACAGGAGACTG	TGCTTCTGCAGCTCCTCAAT
JunB	CT TCC ACC TCG ACG TTT ACA	AAT CGA GTC TGT TTC CAG CAG AA
ATF2	TGGACAAACCATGCCTGTTG	GACCTGGGATTCCTGGAACA
ATF3	GCAGAAAGAGTCGGAGAAGC	TTCTGAGCCCGGACAATACA
cMYC	ACCACCAGCAGCGACTCTGA	TCCAGCAGAAGGTGATCCAGACT
SRC	ACAACACAGAGGGAGACTGG	ACTCCCGTCTGGTGATCTTG
CAPN2	AATGACAACTGCCCAAGCTG	ATCTCCAGGCGGGAATAGTG
CAPN7	CAGGCACTAGACAGAGCAGA	GGAGGTGGCTTTGGCTTAAC
CAPNS1	TGATGGATAGCGACACCACA	ACCTGGGAGTTCACTACTGC
CASP9	GCTTAGGGTCGCTAATGCTG	TGCAAGATAAGGCAGGGTGA
CASP8	GCAGAGGGAACCTGGTACAT	TGTGAAAGTAGGCTGAGGCA
CASP7	AAGTGACAGGTATGGGCGTT	GCAGATCTTGCATCTTGGCA
CASP6	ACCTGCGCAGATAGAGACAA	ACACAAAGCAATCGGCATCT
CASP3	ACTCCACAGCACCTGGTTAT	GTTTCAGCATGGCACAAAGC
CUL3	CGAGCACCATGTCGAATCTG	CATCTTCTCGCACCGGAAAG
CARP1	GGCCATGAAGGGAGAGCTTA	TCATCCTCATCATCGTCGTCA
CARP2	CTTGGAACCAAGCTGCAAGT	TCCCTACTTGGCTCGAACAG

Figure S1

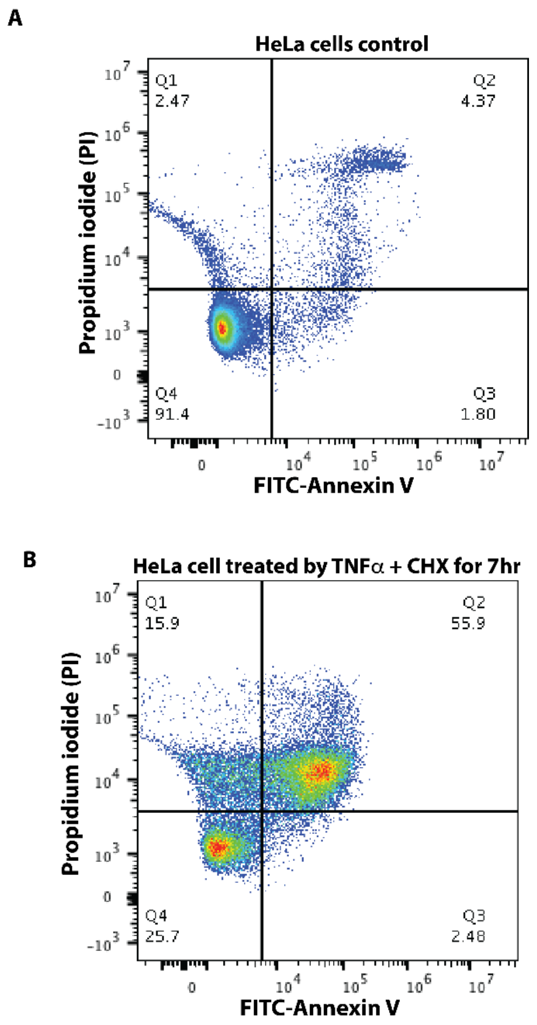

Figure S1. Annexin V and propidium iodide (PI) stain of apoptotic cells.
Hela cells without (A) or with (B) 30ng/ml TNFα plus 10μg/ml CHX for 7hrs were subjected to apoptotic analysis using flow cytometry probed by FITC-Annexin V and PI.
